# Magnetic Catechol-Chitosan with Bioinspired Adhesive Surface: Preparation and Immobilization of ω-Transaminase

**DOI:** 10.1371/journal.pone.0041101

**Published:** 2012-07-17

**Authors:** Kefeng Ni, Xu Zhou, Li Zhao, Hualei Wang, Yuhong Ren, Dongzhi Wei

**Affiliations:** State Key Laboratory of Bioreactor Engineering, New World Institute of Biotechnology, East China University of Science and Technology, Shanghai, China; University of Minho, Portugal

## Abstract

The magnetic chitosan nanocomposites have been studied intensively and been used practically in various biomedical and biological applications including enzyme immobilization. However, the loading capacity and the remained activity of immobilized enzyme based on existing approaches are not satisfied. Simpler and more effective immobilization strategies are needed. Here we report a simple catechol modified protocol for preparing a novel catechol-chitosan (CCS) - iron oxide nanoparticles (IONPs) composites carrying adhesive moieties with strong surface affinity. The ω-transaminase (ω-TA) was immobilized onto this magnetic composite via nucleophilic reactions between catechol and ω-TA. Under optimal conditions, 87.5% of the available ω-TA was immobilized on the composite, yielding an enzyme loading capacity as high as 681.7 mg/g. Furthermore, the valuation of enzyme activity showed that ω-TA immobilized on CCS-IONPs displayed enhanced pH and thermal stability compared to free enzyme. Importantly, the immobilized ω-TA retained more than 50% of its initial activity after 15 repeated reaction cycles using magnetic separation and 61.5% of its initial activity after storage at 4°C in phosphate buffered saline (PBS) for 15 days. The results suggested that such adhesive magnetic composites may provide an improved platform technology for bio-macromolecules immobilized.

## Introduction

Chitosan (CS), a linear amino polysaccharide, is derived from chitin which is found in abundance in nature [1]. CS has been widely applied in food and pharmaceutical industries due to its unique characteristics such as non-toxicity, hydrophilicity, biocompatibility, biodegradability, physiological inertness and high mechanical strength [2]. In recent years, the magnetic CS nanocomposites, as a special functional material, have attracted much attention and been applied in loading of biomolecules [3], biosensor [4], metal adsorption [5], drug/gene delivery [6], and MRI [7].

Generally, the CS-IONPs composites were synthesized using microemulsion polymerization, in situ polymerization and suspension cross-linking method [8]. Several new methods have recently been developed to synthesize CS-IONPs composites using IONPs covalently attached on the amine groups of CS for various applications [9], [10]. However, for immobilization of enzyme onto CS-IONPs composites, although the capacity of enzyme via covalently linking is more than via electrostatic attraction absorption [11], [12], the loading capacity and the remained activity of enzyme are not satisfied as expected using glutaraldehyde as coupling reagent. One main reason may be that glutaraldehyde has an aldehyde group on both ends of the molecular which can simultaneously react with the amine group of CS in the typical glutaraldehyde activation procedure. This side reaction reduces the number of CS-IONPs composites reaction with enzyme. Furthermore, glutaraldehyde can cause denaturation and distortion of most enzymes due to the same reaction between aldehyde groups of glutaraldehyde and amine groups of enzyme.

Herein, inspired by adhesive proteins secreted by marine mussels (*Mytilus edulis*) [13], we developed a new strategy of using catechol (ortho-dihydroxyphenyl) modified chitosan (CCS) to prepare CCS-IONPs composites to form adhesive surface as linking agent for immobilizing enzyme. CCS which containing both catechol and primary amine was synthesized using 3, 4-dihydroxybenzaldehyde to react with the amine groups of CS to form Schiff base in acid solution. Catechol is the functional group of 3, 4-dihydroxy-L-phenylalanine (DOPA) which is abundantly found in the mussel adhesive proteins [14]. It forms strong bonds with various inorganic/organic surfaces in aqueous media [15].In this study, the adhesive CCS-IONPs composites were prepared and used to immobilize ω-TA. We choose ω-TA because it is an important kind of enzyme with a variety of applications in pharmaceutical industry [16], [17]. Enhanced activity and operation stability of ω-TA could be achieved more efficiently and conveniently by immobilization.

## Results

### Synthesis and Characterization of the CCS-IONPs Composites

**Figure 1:**
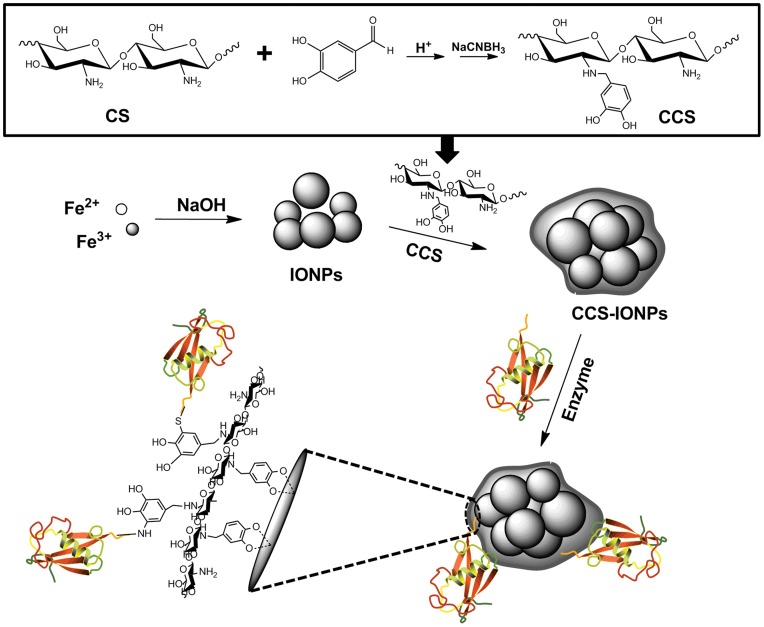
Schematic diagram. Overall strategy for the synthesis of the adhesive CCS-IONPs composites. The CCS was synthesized by Bosch reduction of Schiff base that results from the reaction of CS and 3, 4-dihydroxy benzaldehyde. The adhesive CCS-IONPs composites were prepared by mixing CCS solution with IONPs and adjusting the pH of solution to neutral. Enzyme was immobilized onto the adhesive CCS-IONPs composites via nucleophilic reactions between catechol and enzyme.

Figure 1 illustrates the overall strategy for the adhesive CCS-IONPs composites preparation and ω-TA immobilization. CCS was synthesized by Bosch reduction of Schiff base that results from the reaction of CS and 3, 4-dihydroxy benzaldehyde. The degree of substitution of CS was calculated from the ^1^H-NMR spectrum (Figure S1) of CCS. In this reaction condition, the degree of substitution was 52.2%.

**Figure 2:**
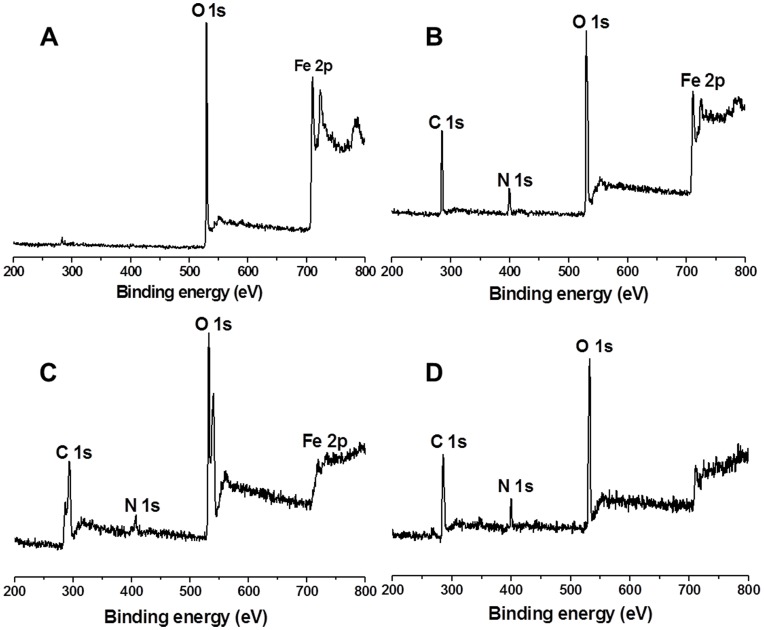
XPS spectra. XPS spectra of particles A: the IONPs; B: the CS-IONPs composites; C: the adhesive CCS-IONPs composites; D: the adhesive CCS-IONPs composites after enzyme immobilized.

X-ray photoelectron spectroscopy (XPS) spectra of the IONPs and composites are shown in Figure 2. Whereas the IONPs contain little carbon and nitrogen content (Figure 2A), the XPS spectrum of CS-IONPs composites contain prominent peaks correlating to C1s (284.5 eV) and N1s (399.5 eV). In the CCS-IONPs composites’ XPS spectrum (Figure 2C), however, the C1s and O1s signals become complex and are peak-fitted into 2 to 3 components, respectively. The C1s peak in 292.5 eV is due to the π-π* transition of aromatic carbons [18], which proves presence of aromatic carbons. The nitrogen-to-carbon (N/C) ratio of CCS-IONPs composites decreases obviously compared with CS-IONPs composites. Additionally, the Fe 2 p peaks become very weak in CCS-IONPs composites compared to IONPs due to surface coating. Further evidence for CCS coating is provided by a shift of the O1s peak from 529.5 eV in IONPs corresponding to iron oxide to 532 eV corresponding to organic oxygen.

**Figure 3:**
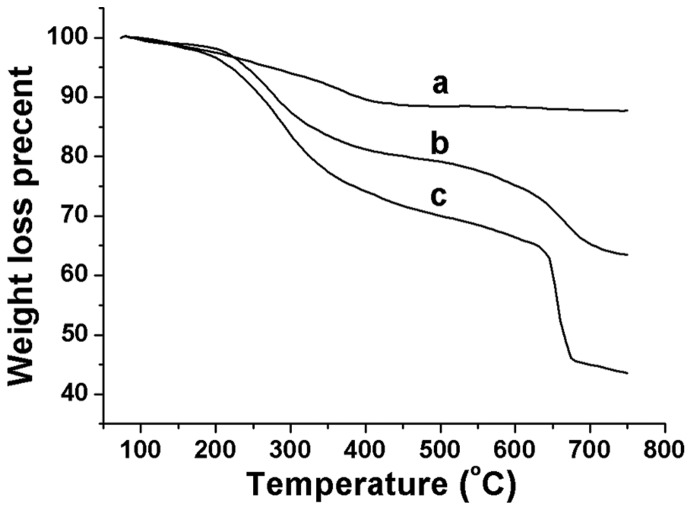
TGA curves. TGA curves of the IONPs (a), the CS-IONPs composites (b) and the adhesive CCS-IONPs composites(c).

Thermogravimetric analysis (TGA) (Figure 3) on the IONPs and composites show that the weight of all samples begins to decrease from 105°C due to the evaporation of bound water. The weight of IONPs becomes stable when above 410°C with a final 12.1% weight loss. However, the weight of the composites continues to decrease due to the evaporation of bound water and thermal decomposition of moisture in the glycosyl of CS. A very quick weight loss is observed from 620°C to 660°C in CCS-IONPs composites which is presumed resulted from the pyrolysis of benzene ring of catechol moieties. The finally weight loss of CS-IONPs composites and CCS-IONPs composites is 36% and 57%, indicating that the mass ratios of CS and CCS is 24% and 45% in composites, respectively.

**Figure 4:**
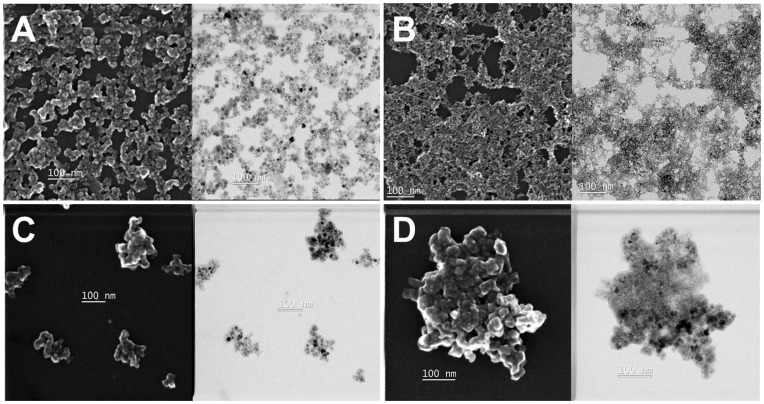
SEM and TEM spectra. SEM (left) and TEM (right) images of A: the IONPs; B: the CS-IONPs composites; C: the adhesive CCS-IONPs composites; D: the adhesive CCS-IONPs composites after enzyme immobilization.

**Figure 5:**
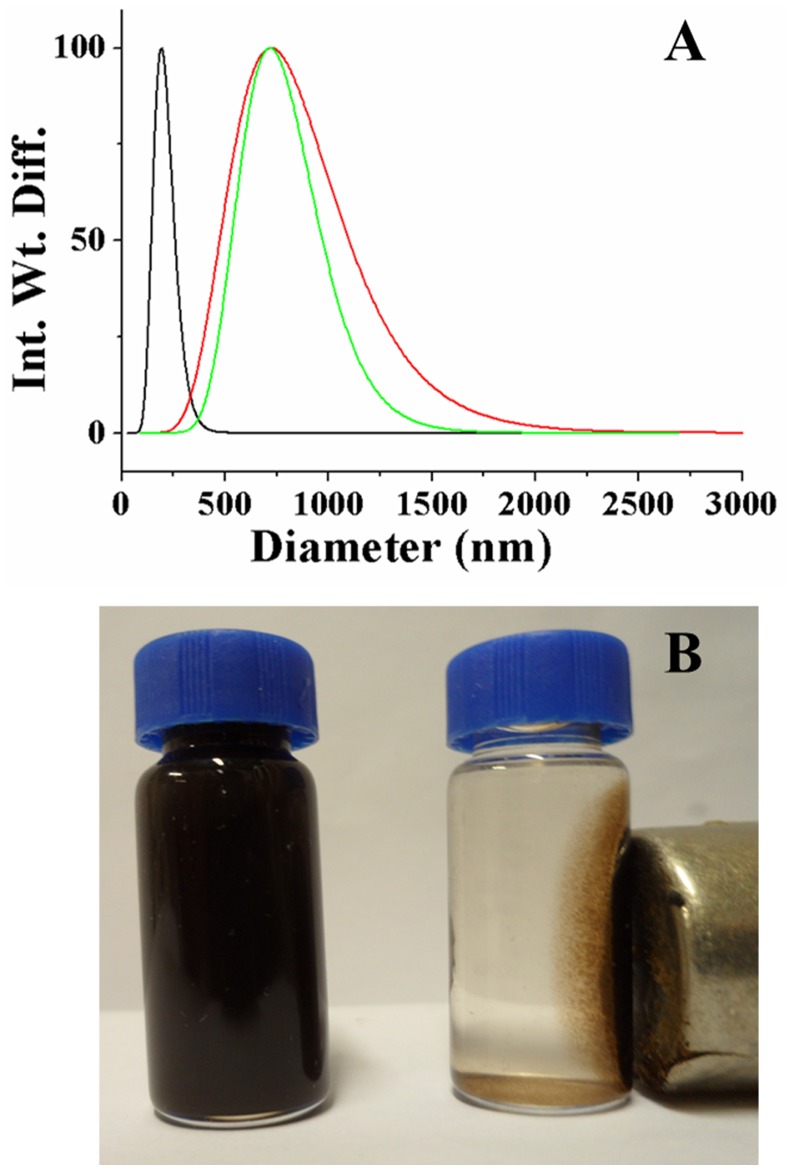
Particle size distributions and magnetic property of the composites. Particle size distributions of IONPs (black), CS-IONPs (red) and CCS-IONPs (green) in ultrapure water (A). Particle sizes were determined by dynamic light scattering, the composites were dispersed at a concentration of 0.1 mg/ml in ultrapure water with ultrasonication for 15 minutes. Dispersion and magnetic property of the CCS-IONPs composites (B). Photographs of an aqueous suspension of the CCS-IONPs composites before (left) and after (right) magnetic isolation.

The size and morphology of IONPs and composites were observed by scanning electron microscopy (SEM) and transmission electron microscopy (TEM) (Figure 4). SEM and TEM images show that the CS-IONPs composites have a porous framework that the IONPs are well dispersed and cross-linked in the CS without any indications of massive agglomeration. Compared to the CS-IONPs composites, the morphology of the CCS-IONPs composites is obviously different. The IONPs agglomerate and are encapsulated tightly by CCS. TEM images show that the IONPs were composed of clusters of nanoparticles with sizes ranging from 5 nm to 10 nm. The particle size distribution of the IONPs and composites was determined by dynamic light scattering (DLS) (Figure 5A). The average size of the CS-IONPs and CCS-IONPs clusters is about 750 nm, larger than as-synthesized IONPs clusters (average diameter 170 nm).

The CCS-IONPs composites exhibit highly responsive to a magnetic field (Figure 5B), the solution became clear within one minute as a result of movement of the CCS-IONPs composites towards a magnet. The composites were re-suspended in water after removing the magnet followed by agitation. These demonstrate that CCS-IONPs composites disperse well in water and can be simply and rapidly separated from solution by a magnet.

### Immobilization of ω-TA onto the CCS-IONPs Composites

XPS spectrum of the ω-TA immobilized CCS-IONPs composites (Figure 2D) shows an increase in N/C ratio (0.153) compared to CCS-IONPs (0.038). However, the morphology of the CCS-IONPs composites shows no any change after immobilization of ω-TA (Figure 4D).

Table 1 summarizes the immobilization efficiency and the specific activity of the ω-TA immobilized onto the composites. The CCS-IONPs display higher enzyme loading capacity than IONPs and CS-IONPs. The amount of ω-TA immobilized onto CCS-IONPs composites was 681.7 mg/g, 3.5-fold of that onto IONPs and over 6-fold of that onto CS-IONPs composites. The specific activity of ω-TA immobilized on CCS-IONPs composites was 7.79 U/mg which retained 87.5% of the initial activity of the free ω-TA (8.64 U/mg).

**Table 1:** The immobilization efficiency of the composites and the specific activity of the immobilized enzyme.

Composites	Bound enzyme (mg/g)	Activity (U/mg carrier particle)	Specific activity^a^ (U/mg)	Activity retention (%)
IONPs	195.3±12.4	1.63±0.05	8.35±0.4	93±3.4
CS-IONPs	107.3±6.9	0.92±0.06	8.54±0.07	95.7±2.4
CCS-IONPs	681.7±11.6	5.31±0.18	7.79±0.25	87.5±0.6

a Specific activity of free ω-TA was 8.64 U/mg.

### Stability and Reusability of the Immobilized ω-TA

**Figure 6:**
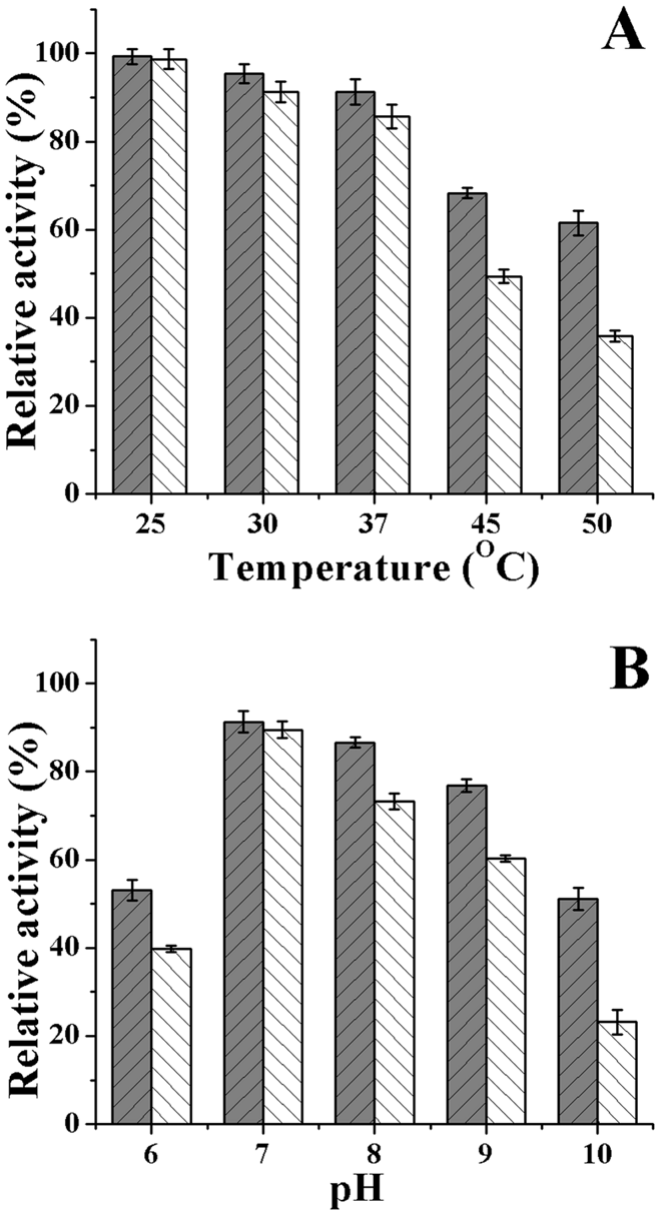
The effects of pH and temperature on the activity of free (white bar) and immobilized enzyme (grey bar). The effect of temperature on free and immobilized enzyme (A). The samples were pre-incubated in PBS at temperature ranging from 25 to 50°C for 3 hours. The effect of pH on free and immobilized enzyme (B). The samples were pre-incubated at 4°C in buffer at pH ranging from 6 to 10 for 24 hours.

The effects of pH and temperature on the activity of free and immobilized ω-TA are shown in Figure 6. The activity of both free and immobilized ω-TA is mildly affected at temperatures below 37°C (Figure 6A). However, the relative activity of free ω-TA decreased significantly above 37°C which retained 36% of the initial activity at 50°C, whereas the immobilized ω-TA retained as high as 62%.

Compared to the free ω-TA, more activity of the immobilized ω-TA was retained after incubated at a wider pH range. As shown in Figure 6B, the activity of the immobilized ω-TA decreased slightly at pH 7–9, and retained 53% and 51% of initial activity after 24 hours incubation at pH 6 and 10, respectively. However, the activity of the free ω-TA has a noticeable reduction after 24 hours incubation at pH 8 and pH 9 which retained 73% and 60% of the initial activity. In addition, only 40% and 23% of the initial activity was retained at pH 6 and 10 after 24 hours incubation.

**Figure 7:**
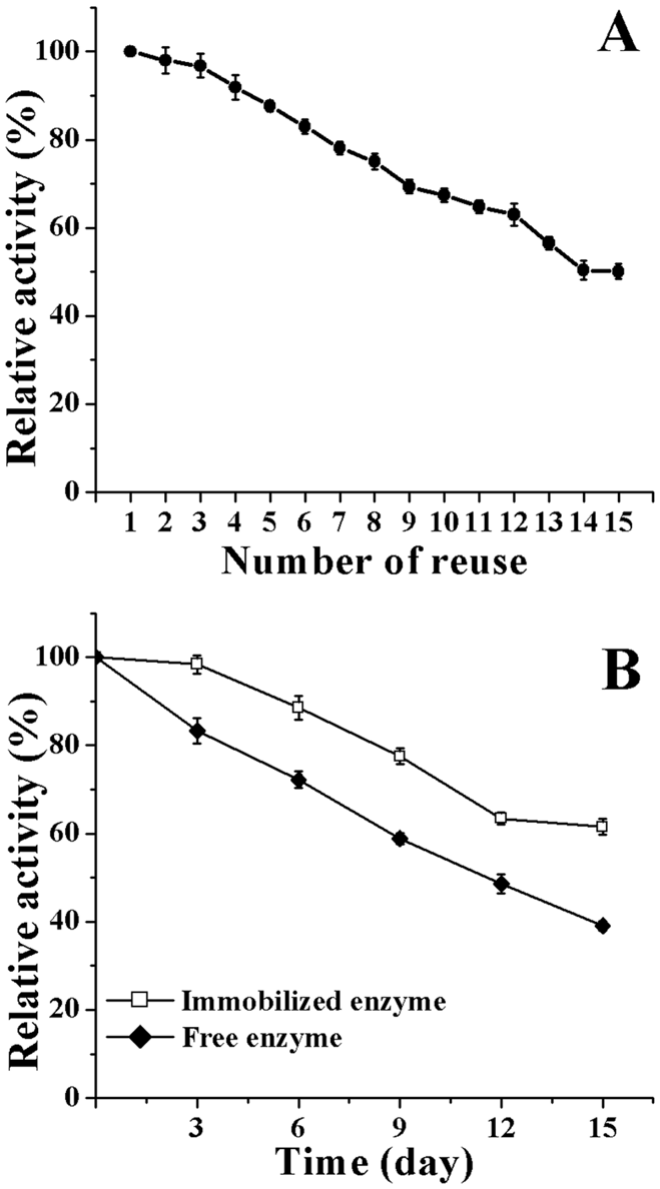
Reusability and storage stability of the immobilized ω-TA. Changes in the activity of immobilized enzyme after multiple cycles of magnetic isolation and reuse (A). The activity of immobilized enzyme after multiple cycles of magnetic isolation and reuse at 37°C (the initial activity of immobilized ω-TA defined as 100%). The total elapsed time of the experiment was less than 8 hours. Storage stability of the free and immobilized enzyme (B). The samples were stored at 4°C in PBS for 15 days, followed by determine the residual activity every 3 days.

Figure 7A shows the variation of activity of the ω-TA immobilized on CCS-IONPs composites after multiple reusing by magnetic separation. Although the activity of the immobilized ω-TA decreased after cycles, more than 50% of its initial activity was still retained after 15 cycles. The storage stability of the free and immobilized ω-TA is shown in Figure 7B. The immobilized ω-TA retained more than 60% of its initial activity after stored at 4°C in PBS for 15 days, whereas the free ω-TA only retained 39% of its initial activity.

## Discussion

The synthetic procedure of CCS-IONPs composites and ω-TA immobilization are illuminated as Figure 1. ^1^H-NMR analysis (Figure S1) proves that the catechol monomer is introduced to CS with a substitution of 52.2%. XPS analysis shows that the surface chemical composition is altered among these materials. The carbon and nitrogen peak on the CCS-IONPs composites confirm the present of CCS coating. The increasing of N/C ratio 0.038 to 0.153 after ω-TA immobilization indicates that ω-TA is immobilized onto the surface of the CCS-IONPs composites. The results of TGA analysis further confirm the conclusion that CCS is successfully coated on IONPs. The composites have magnetic property and can be easily separated by magnet.

SEM and TEM images (Figure 4) show that the morphology of the CCS-IONPs composites is different from that of CS-IONPs composites. The reason is presumed that when water was evaporated during the drying procedure, catechol bounded on CS damaged the stability network stereo-structure which formed via electrostatic interactions and hydrogen bound among catechol, CS, IONPs and water in aqueous environment. Catechol might be self-polymerized or covalently linked with the remained amine groups of CCS to cause CCS-IONPs composites cross-linked and twisted, finally formed tightly aggregation during the evaporation of the bound water. The result is similar to the process of drying of melanin. It is observed that the process of drying produces melanin aggregation [19].

The efficiency of ω-TA immobilization using the adhesive composites was investigated. Compared to the IONPs and the CS-IONPs composites, the adhesive CCS-IONPs composites display better enzyme loading capacity (681.7 mg/g), which is the higher than present reports [20], [21]. The high loading efficiency is attributed to the strong affinity anchor from catechol which exhibited reactivity toward amine and thiol groups [15]. This may also be resulted from the high surface area due to the nano-porous morphology of the composites. Although we did not observe morphology of the adhesive composites in liquid state, the SEM and TEM images (Figure 4B and 4C) show that there are nano-porous in the aggregation after dried.

The stability of immobilized ω-TA was investigated in various pH and temperatures. Compared to free ω-TA, the stability of immobilized ω-TA increase significantly, retaining 25% and 68% of its initial activity even after 60 hours incubation in pH 5 and 9, respectively. The stability increase after immobilization may result from multipoint covalent linking between ω-TA and the CCS-IONPs composites, leading to less denaturation of ω-TA. The reusability and storage stability of immobilized enzymes are very important properties in their application, especially in industrial applications. As shown in Figure 7, 51% of its initial activity was still retained after 15 cycles, and more than 60% of its initial activity was retained after stored at 4°C in PBS for 15days which is significantly higher than the free enzyme retained (39%) at the same condition. The results indicate that the ω-TA immobilized onto the adhesive CCS-IONPs composites has good stability, reusability and magnetic recovery. The decrease of activity may be caused by the aggregation and the leakage of the particles, the denaturation of the protein and the gradually ω-TA detachment from the carriers.

## Materials and Methods

### Materials

The recombinant *E.coli* strain harboring ω-TA gene from *Burkholderia cenocepacia* J2315 was obtained in our laboratory. Chitosan (low molecular weight), 3, 4-dihydroxy benzaldehyde, sodium cyanoborohydride (NaBH_3_CN) and isopropyl-β-D-thiogalactopyranoside (IPTG) were purchased from Sigma-Aldrich (USA). (S)-α-methyl benzylamine ((S)-α-MBA), sodium pyruvate, pyridoxal-5′-phosphate (PLP) were purchased from Adamas (Switzerland). Other analytical grade chemicals were purchased from SCRC (China).

### Preparation of CCS-IONPs Composites

Figure 1 illustrates the synthesis of CCS-IONPs composites. CCS was prepared by Bosch reduction according to the method reported earlier [22] with some modifications. Briefly, 0.1 g CS powder was dissolved in 20 ml 1% acetic acid solution and then 5 ml DMF contain 0.3 g 3, 4-dihydroxy benzaldehyde was added. After magnetic stirred at room temperature for 6 hours, 40 ml ethanol was added and pH was adjusted to 6.5 using 1 M NaOH. Precipitation (Schiff base) was observed and filtered, washed with water and ethanol to remove residual reagent. The Schiff base was suspended in 20 ml water and 0.3 g NaBH_3_CN was added. After stirred at room temperature for 6 hours, 40 ml ethanol was added. The product was filtered and washed with water and ethanol, and then dried at room temperature in vaccum. CCS was characterized by ^1^H-NMR. The degree of substitution for catechol derivative was calculated from the ^1^H-NMR spectrum.

IONPs were synthesized by co-precipitation method [23]. Generally, 10 mM FeCl_3_ and 5 mM FeCl_2_ were dissolved in 100 ml ultrapure water under nitrogen, the pH of the solution was adjusted to 9.5 using 1 M NaOH solution. After stirred for 2 hours at 40°C, IONPs were collected and washed several times with ultrapure water using a magnetite, and dispersed in 100 ml ultrapure water under ultrasonication for 15 min.

The CCS-IONPs composites were prepared by adding 50 ml IONPs (50 mg) solution to 50 ml 1% acetic acid solution contain 100 mg CCS under vigorous stirring, then the pH of mixture was adjusted to 7.0 using 1 M NaOH solution. After stirred for 1 hour at room temperature, CCS-IONPs composites were separated using magnetite and washed with ultrapure water several times. The CCS-IONPs composites were stored at 4°C. The CS-IONPs composites were prepared by the same method using CS instead of CCS.

### Characterization of IONPs and CCS-IONPs Composites

XPS spectra were obtained using an Omicron ESCALAB (Omicron, Taunusstein, Germany) with a monochromatic Al Kα (1486.8 eV) 300-W X-ray source, a flood gun to counter charging effects, and ultrahigh vacuum (∼10^−9^ torr). The takeoff angle was fixed at 45°. Substrates were mounted on sample studs by means of double-sided adhesive tape. All binding energies were calibrated using the C (1 s) carbon peak (284.5 eV). Thermo gravimetric analysis (TGA) was carried out on a SDT 2960 model from TA Instruments. All samples were dried under vacuum overnight to remove moisture before analysis and were performed under nitrogen flow with a heating rate of 10°C/min. Scanning electron microscope (SEM) and transmission electron microscopy (TEM) images were acquired on a Hitachi HD2300 electron microscope (Hitachi, Japan) operated at 200 kV. The specimens were prepared by casting drops of dilute dispersion of composites aqueous solution on 200-mesh carbon coated copper grids and air-drying. Dynamic light scattering (DLS) experiments were carried out on a Nicomp 380 ZLS (PPS, USA). The CS-IONPs, CCS-IONPs and IONPs were dispersed at a concentration of 0.1 mg/ml in ultrapure water with ultrasonication for 15 minutes.

### Preparation of ω-TA

The recombinant *E.coli* strain was cultured in LB-ampicillin at 37°C. When the optical density of cells in culture at 600 nm reached 0.6, IPTG was added to 0.1 mM. After incubated at 25°C for 16 hours, the cells were harvested by centrifugation (10 min, 4°C, 8000 rpm), washed with PBS and re-suspended in the same buffer. After ultrasonicated at 4°C for 15 min in ultrasonic cell disruptor (JY92-II, SCIENTZ, China), the raw enzyme solution of ω-TA was obtained after centrifugation at 10000 rpm for 15 min at 4°C. The supernatant was loaded on HisTrap™ FF crude column (Pharmacia, USA). The proteins were eluted with imidazole and desalted by ultrafiltration. The SDS-PAGE was performed to check the results of expression and purification (Figure S2). The specific activity of the ω-TA was 8.64 U/mg measured by the assay described in activity assay section.

### Immobilization of ω-TA

The fresh CCS-IONPs composites prepared previously were dispersed in PBS under ultrasonication for 15 min. The immobilization of ω-TA onto CCS-IONPs composites was carried out by directly adding 1 ml enzyme solution (contain 1 mg ω-TA) to 1 ml CCS-IONPs composites (1 mg/ml) in PBS. After shaken at 4°C for 3 hours, the ω-TA loaded precipitates were collected and washed 3 times with ultrapure water and stored at 4°C prior to be used. The protein concentration of enzyme solution before and after immobilization was determined using the Bradford method [24]. The difference in protein concentration was used to calculate the loading of ω-TA onto the CCS-IONPs composites. Immobilization of ω-TA onto CS-IONPs and IONPs were carried out by the same method using IONPs or CS-IONPs instead of CCS-IONPs.

### Activity Assay of Free and Immobilized ω-TA

The enzymatic activities of free and immobilized ω-TA were measured by determining the concentration of acetophenone that results from the reaction showed in Figure S3 at 37°C in phosphate buffer (pH 8.0, 50 mM). The typical reaction volume was 1 ml of 10 mM (S)-α-methyl benzylamine ((S)-α-MBA) and 10 mM sodium pyruvate with 0.1 mM PLP as co-enzyme. After reacted for 10 min at 37°C, the reaction was stopped by addition of 200 µl 1 M HCl solution. The reaction mixtures were analyzed by HPLC (1100 Series Agilent) using a C18 reverse phase column (ODS2, 4.6 mm×250 mm, 5 µm, Thermo) at 210 nm and 245 nm with isocratic elution of 50% methanol and 50% phosphate buffer (pH 4.0, 20 mM). One unit of enzyme is defined as the amount of ω-TA which catalyzed the formation of 1 µmol acetophenone per minute under the assay condition.

All activity measurements were carried out at least three times and the experimental error was less than 5%.

### Stability and Reusability of Immobilized ω-TA

The effect of pH on the activity of the free and immobilized ω-TA was determined by pre-incubated at 4°C in buffer at pH ranging from 6 to 10 for 24 hours, followed by determination of enzyme activity at pH 8.0 as described in activity assay section. For the thermal stability, the immobilized ω-TA was pre-incubated in phosphate buffer (pH 8.0, 50 mM) at temperature ranging from 25 to 50°C for 3 hours, followed by measurement of the residual enzyme activity at 37°C as described above.

To determine the storage stability of the enzyme, the free and immobilized ω-TA was stored in PBS at 4°C, followed by measurement of the residual enzyme activity every 3 days as described above. The reusability of the immobilized ω-TA under conditions of repeated magnetic isolation and reuse was investigated under the same conditions as described in activity assay section. After each run, the immobilized ω-TA was collected and washed twice with ultrapure water using a magnetite to remove any remaining substrate and product species before the next cycle. The residual activity of the immobilized ω-TA after each cycle was normalized to the initial value (the initial activity was defined as 100%).

## Supporting Information

Figure S1
**^1^H-NMR spectra.** ^1^H-NMR of CCS was detected on Varian INOVA 400 ^1^H-NMR spectroscopy (USA) using D_2_O contain 1% DCl as solvent. The degree of substitution of CS was calculated from ^1^H-NMR spectrum of CCS by comparing the intensity of the C-2 proton signal of sugar with that of the aromatic protons (Equation 1). 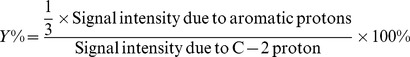

(TIF)

Figure S2 **SDS-PAGE analysis.** Lane 1 for the standard protein marker, lane 2 for the raw enzyme solution and lane 3 for the purified ω-TA.
(TIF)

Figure S3 **Reaction scheme.** Reaction scheme of ω-TA between (S)-α-MBA and sodium pyruvate. The reaction volume was 1 ml of 10 mM (S)-α-methyl benzylamine ((S)-α-MBA) and 10 mM sodium pyruvate with 0.1 mM PLP as co-enzyme.
(TIF)

## References

[1] Kim JY, Lee JK, Lee TS, Park WH (2003). Synthesis of chitooligosaccharide derivative with quaternary ammonium group and its antimicrobial activity against Streptococcus mutans.. International journal of biological macromolecules.

[2] Orrego C, Salgado N, Valencia J, Giraldo G, Giraldo O (2010). Novel chitosan membranes as support for lipases immobilization: Characterization aspects.. Carbohydrate Polymers.

[3] Wu Y, Wang Y, Luo G, Dai Y (2009). In situ preparation of magnetic Fe3O4-chitosan nanoparticles for lipase immobilization by cross-linking and oxidation in aqueous solution.. Bioresource technology.

[4] Kaushik A, Khan R, Solanki PR, Pandey P, Alam J (2008). Iron oxide nanoparticles-chitosan composite based glucose biosensor.. Biosensors and Bioelectronics.

[5] Tran HV, Tran LD, Nguyen TN (2010). Preparation of chitosan/magnetite composite beads and their application for removal of Pb (II) and Ni (II) from aqueous solution.. Materials Science and Engineering: C.

[6] Zhu L, Ma J, Jia N, Zhao Y, Shen H (2009). Chitosan-coated magnetic nanoparticles as carriers of 5-Fluorouracil: Preparation, characterization and cytotoxicity studies.. Colloids and Surfaces B: Biointerfaces.

[7] Ge Y, Zhang Y, He S, Nie F, Teng G (2009). Fluorescence Modified Chitosan-Coated Magnetic Nanoparticles for High-Efficient Cellular Imaging.. Nanoscale research letters.

[8] Zhang L, Zhu X, Sun H, Chi G, Xu J (2010). Control synthesis of magnetic Fe3O4-chitosan nanoparticles under UV irradiation in aqueous system.. Current Applied Physics.

[9] Yuan Q, Venkatasubramanian R, Hein S, Misra R (2008). A stimulus-responsive magnetic nanoparticle drug carrier: Magnetite encapsulated by chitosan-grafted-copolymer.. Acta Biomaterialia.

[10] Zhao DL, Wang XX, Zeng XW, Xia QS, Tang JT (2009). Preparation and inductive heating property of Fe3O4-chitosan composite nanoparticles in an AC magnetic field for localized hyperthermia.. Journal of Alloys and Compounds.

[11] Orrego CE, Valencia JS (2009). Preparation and characterization of chitosan membranes by using a combined freeze gelation and mild crosslinking method.. Bioprocess and biosystems engineering.

[12] Orrego CE, Valencia JS, Zapata C (2009). Candida rugosa Lipase Supported on High Crystallinity Chitosan as Biocatalyst for the Synthesis of 1-Butyl Oleate.. Catalysis letters.

[13] Lee H, Dellatore SM, Miller WM, Messersmith PB (2007). Mussel-inspired surface chemistry for multifunctional coatings.. Science.

[14] Lee H, Rho J, Messersmith PB (2009). Facile conjugation of biomolecules onto surfaces via mussel adhesive protein inspired coatings.. Advanced Materials.

[15] Lee H, Scherer NF, Messersmith PB (2006). Single-molecule mechanics of mussel adhesion.. Proceedings of the National Academy of Sciences.

[16] Koszelewski D, Tauber K, Faber K, Kroutil W (2010). ω-Transaminases for the synthesis of non-racemic α-chiral primary amines.. Trends in Biotechnology.

[17] Truppo MD, Rozzell JD, Turner NJ (2009). Efficient production of enantiomerically pure chiral amines at concentrations of 50 g/L using transaminases.. Organic Process Research & Development.

[18] Stepanow S, Strunskus T, Lingenfelder M, Dmitriev A, Spillmann H (2004). Deprotonation-driven phase transformations in terephthalic acid self-assembly on Cu (100).. The Journal of Physical Chemistry B.

[19] Zhao H, Sun C, Stewart RJ, Waite JH (2005). Cement proteins of the tube-building polychaete Phragmatopoma californica.. Journal of Biological Chemistry.

[20] Yi SS, Lee C, Kim J, Kyung D, Kim BG (2007). Covalent immobilization of ω-transaminase from Vibrio fluvialis JS17 on chitosan beads.. Process Biochemistry.

[21] Jiang Y, Guo C, Xia H, Mahmood I, Liu C (2009). Magnetic nanoparticles supported ionic liquids for lipase immobilization: Enzyme activity in catalyzing esterification.. J Mol Catal B Enzym.

[22] Desbrieres J, Martinez C, Rinaudo M (1996). Hydrophobic derivatives of chitosan: characterization and rheological behaviour.. International journal of biological macromolecules.

[23] Gupta AK, Gupta M (2005). Synthesis and surface engineering of iron oxide nanoparticles for biomedical applications.. Biomaterials.

[24] Bradford MM (1976). A rapid and sensitive method for the quantitation of microgram quantities of protein utilizing the principle of protein-dye binding.. Analytical Biochemistry.

