# Supplementary figures and images for: Magnetic Catechol-Chitosan with Bioinspired Adhesive Surface: Preparation and Immobilization of ω-Transaminase
Source: PLoS One. 2012 Jul 17;7(7):e41101. doi: 10.1371/journal.pone.0041101 (PMC3398885; doi:10.1371/journal.pone.0041101)

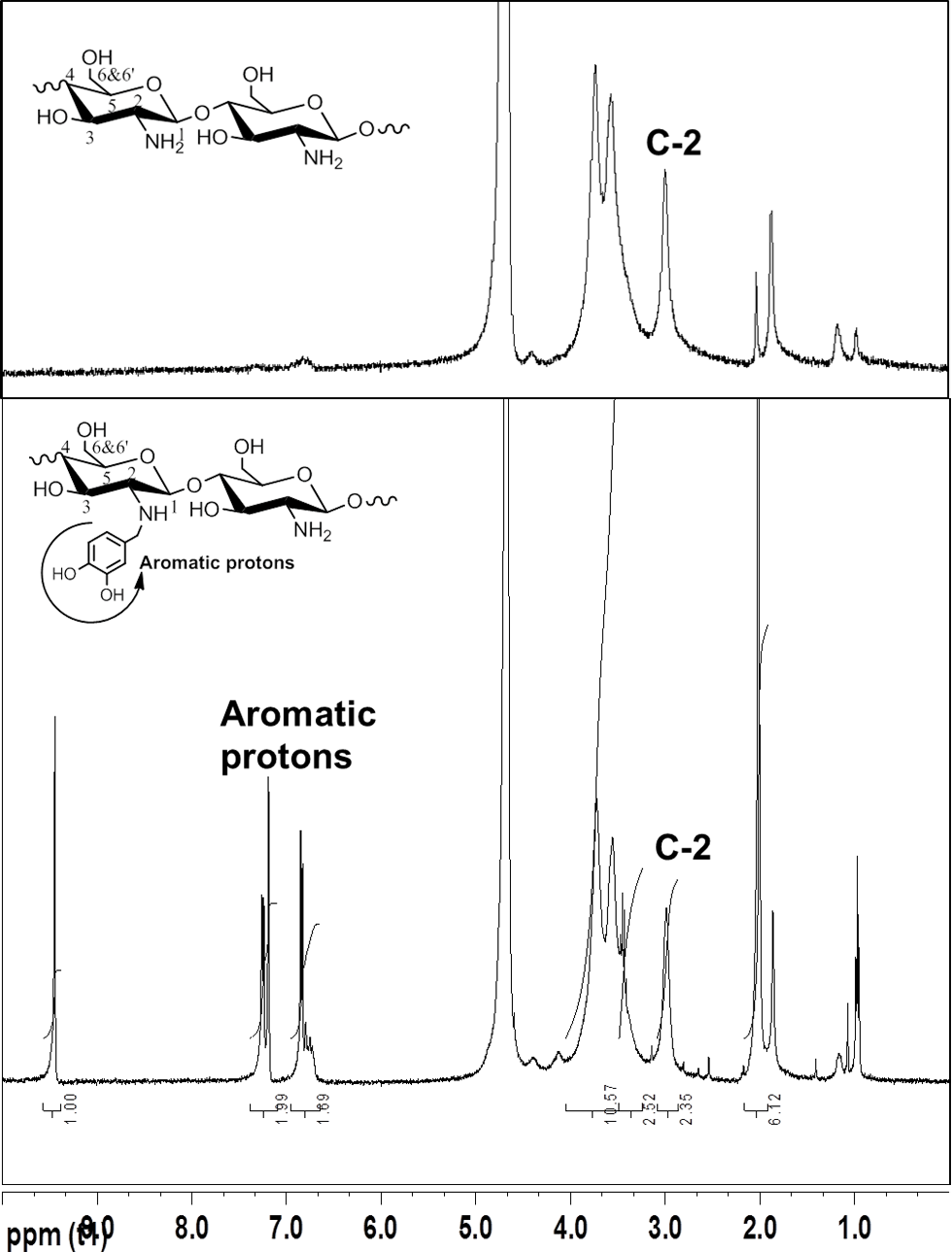

Supplement: Figure S1 — 1H-NMR spectra. 1H-NMR of CCS was detected on Varian INOVA 400 1H-NMR spectroscopy (USA) using D2O contain 1% DCl as solvent. The degree of substitution of CS was calculated from 1H-NMR spectrum of CCS by comparing the intensity of the C-2 proton signal of sugar with that of the aromatic protons (Equation 1). (TIF) [file pone.0041101.s001.tif]

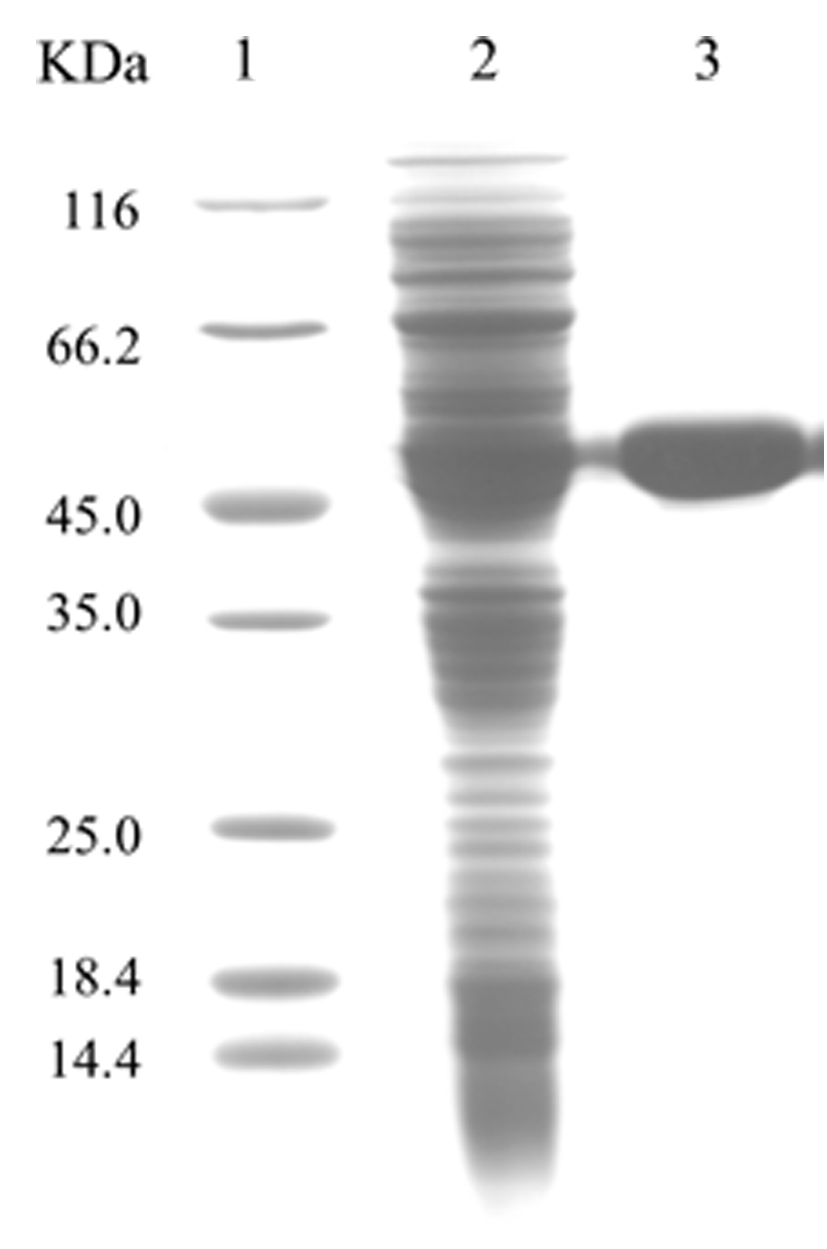

Supplement: Figure S2 — SDS-PAGE analysis. Lane 1 for the standard protein marker, lane 2 for the raw enzyme solution and lane 3 for the purified ω-TA. (TIF) [file pone.0041101.s002.tif]
